# Epidemiology of Usutu Virus: The European Scenario

**DOI:** 10.3390/pathogens9090699

**Published:** 2020-08-26

**Authors:** Tatjana Vilibic-Cavlek, Tamas Petrovic, Vladimir Savic, Ljubo Barbic, Irena Tabain, Vladimir Stevanovic, Ana Klobucar, Anna Mrzljak, Maja Ilic, Maja Bogdanic, Iva Benvin, Marija Santini, Krunoslav Capak, Federica Monaco, Eddy Listes, Giovanni Savini

**Affiliations:** 1 Department of Virology, Croatian Institute of Public Health, 10000 Zagreb, Croatia; irena.tabain@hzjz.hr (I.T.); maja.bogdanic11@gmail.com (M.B.); 2 School of Medicine, University of Zagreb, 10000 Zagreb, Croatia; anna.mrzljak@gmail.com; 3 Department for Virology, Scientific Veterinary Institute, 21000 Novi Sad, Serbia; tomy@niv.ns.ac.rs; 4 Poultry Center, Croatian Veterinary Institute, 10000 Zagreb, Croatia; v_savic@veinst.hr; 5 Department of Microbiology and Infectious Diseases with Clinic, Faculty of Veterinary Medicine, University of Zagreb, 10000 Zagreb, Croatia; ljubo.barbic@vef.hr (L.B.); vladimir.stevanovic@vef.hr (V.S.); ibenvin@vef.hr (I.B.); 6 Department of Epidemiology, Andrija Stampar Teaching Institute of Public Health, 10000 Zagreb, Croatia; ana.klobucar@stampar.hr; 7 Department of Medicine, Merkur University Hospital, 10000 Zagreb, Croatia; 8 Department of Epidemiology, Croatian Institute of Public Health, 10000 Zagreb, Croatia; maja.ilic@hzjz.hr; 9 Department for Intensive Care Medicine and Neuroinfectology, University Hospital for Infectious Diseases “Dr Fran Mihaljevic”, 10000 Zagreb, Croatia; marijasantini.ms@gmail.com; 10 Environmental Health Department, Croatian Institute of Public Health, 10000 Zagreb, Croatia; kcapak@hzjz.hr; 11 OIE Reference Center for West Nile Disease, Istituto Zooprofilattico Sperimentale “G. Caporale”, 64100 Teramo, Italy; f.monaco@izs.it (F.M.); g.savini@izs.it (G.S.); 12 Laboratory for Diagnostics, Croatian Veterinary Institute, Regional Institute Split, 21000 Split, Croatia; e.listes.vzs@veinst.hr

**Keywords:** Usutu virus, epidemiology, Europe, “One Health”

## Abstract

Usutu virus (USUV) is an emerging arbovirus isolated in 1959 (Usutu River, Swaziland). Previously restricted to sub-Saharan Africa, the virus was introduced in Europe in 1996. While the USUV has received little attention in Africa, the virus emergence has prompted numerous studies with robust epidemiological surveillance programs in Europe. The natural transmission cycle of USUV involves mosquitoes (vectors) and birds (amplifying hosts) with humans and other mammals considered incidental (“dead-end”) hosts. In Africa, the virus was isolated in mosquitoes, rodents and birds and serologically detected in horses and dogs. In Europe, USUV was detected in bats, whereas antibodies were found in different animal species (horses, dogs, squirrels, wild boar, deer and lizards). While bird mortalities were not reported in Africa, in Europe USUV was shown to be highly pathogenic for several bird species, especially blackbirds (*Turdus merula)* and great gray owls (*Strix nebulosa*). Furthermore, neurotropism of USUV for humans was reported for the first time in both immunocompromised and immunocompetent patients. Epizootics and genetic diversity of USUV in different bird species as well as detection of the virus in mosquitoes suggest repeated USUV introductions into Europe with endemization in some countries. The zoonotic potential of USUV has been reported in a growing number of human cases. Clinical cases of neuroinvasive disease and USUV fever, as well as seroconversion in blood donors were reported in Europe since 2009. While most USUV strains detected in humans, birds and mosquitoes belong to European USUV lineages, several reports indicate the presence of African lineages as well. Since spreading trends of USUV are likely to continue, continuous multidisciplinary interventions (“One Health” concept) should be conducted for monitoring and prevention of this emerging arboviral infection.

## 1. Introduction

**Figure 1:**
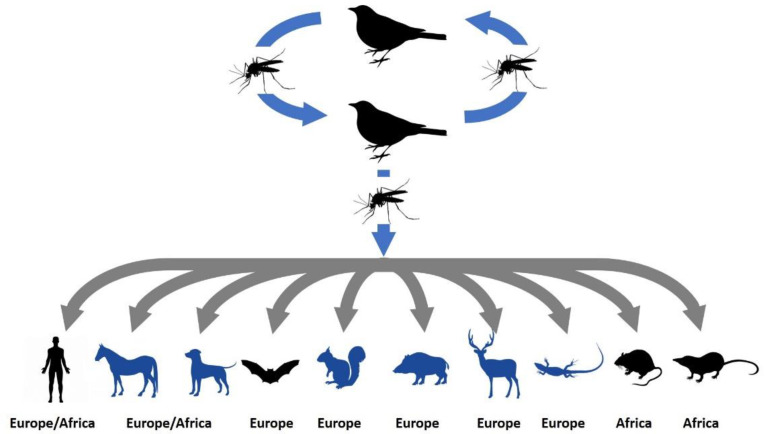
Usutu virus (USUV) transmission cycle involves birds (amplifying hosts) and mosquitoes (vectors). Infection can be transmitted to humans and horses which are generally considered incidental or “dead-end” hosts. USUV isolation/detection (black symbols) and serologic evidence (blue symbols) were reported in different animal species, expanding the incidental host range.

Usutu virus (USUV) is an emerging arbovirus that belongs to the family *Flaviviridae*, genus *Flavivirus*, Japanese encephalitis serocomplex. Similar to other flaviviruses, USUV is a spherical, small, enveloped virus with a single stranded positive-sense RNA genome of ~12 kb. Phylogenetic analyses of the NS5 gene have shown that USUV strains clustered into eight genetic lineages: three African (Africa 1–3) and five European (Europe 1–5) [1]. In nature, USUV is maintained in a bird–mosquito–bird cycle, however the virus or antibodies detection were reported sporadically in humans, horses and other mammals (Figure 1).

USUV was isolated in 1959 from *Culex neavei* mosquito caught near the Usutu River in Swaziland [2]. After that, the virus was confined to Africa with only few human cases with fever, rash and jaundice reported [3]. In Africa, the USUV host range includes mosquitoes, birds, equids and dogs [4,5]. The virus was also isolated and sequenced from five asymptomatic small mammals in Senegal that belong to two rodent species (black rat; *Rattus rattus* and multimammate rat; *Mastomys natalensis*) and a single species of shrew (*Crocidura* sp.) (Figure 1) [6].

A retrospective analysis of archived tissue samples originating from a bird die-off showed that USUV emerged in Europe in 1996 (Tuscany region, Italy), five years before the advent of USUV-associated bird deaths in Austria which has been generally assumed as the starting point of the virus spread in Europe [7]. In 2001, the virus caused the first large outbreak in several bird species in the region of Vienna (Austria) [8]. In the following years, continuous geographic expansion of the USUV in Europe has been shown by reports of epizootics or small outbreaks as well as serologic detection in different wild and captive bird species [9,10,11,12]. In addition, the virus was detected in different native (mainly *Culex pipiens*) and invasive mosquito species (*Aedes albopictus*, *Ae. japonicus*) [13,14,15,16,17,18]. USUV RNA was also found in bats (*Pipistrellus pipistrellus*) in Germany and Belgium [19,20]. Moreover, USUV antibodies were sporadically detected in horses [21,22,23], dogs [24], squirrels [25], wild boar, roe deer [26] and lizards [27] (Figure 1), expanding the USUV host range (Figure 1), however these species are considered incidental hosts. 

The zoonotic potential of USUV has been reported in a limited number of human cases. Few cases of neuroinvasive disease and USUV fever, as well as seroconversion in blood donors were reported in Europe since 2009 [11,28,29,30,31,32,33]. Phylogenetic analyses showed that the majority of USUV strains detected in humans, birds and mosquitoes belong to European USUV lineages, however several reports indicated the presence of African lineages as well [18,34,35,36]. 

**Figure 2:**
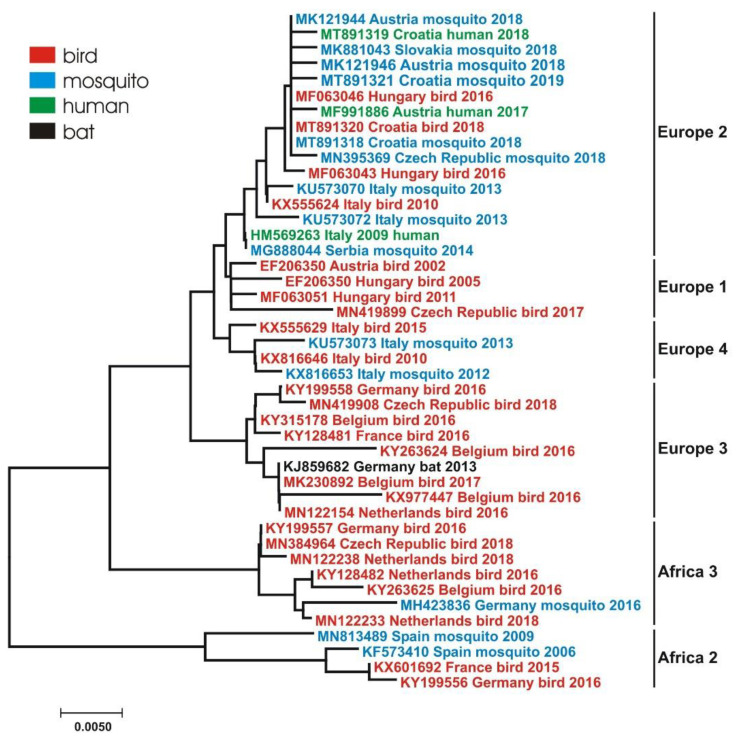
Neighbor-joining tree of USUV sequences based on 495 nucleotides of the NS5 gene illustrating phylogenetic and host diversity of USUV strains detected in European countries. Taxon information includes the GenBank accession number, country in which the virus was detected, host and isolation/detection year. Color codes for different hosts are shown in the upper left corner. USUV genetic lineages are indicated on the right. Scale bar indicates the mean number of nucleotide substitutions per site.

The USUV diversity in Europe appeared in the last decade, however, the phylogenies suggest a long-term virus circulation in this region [37]. The presence of USUV was documented by virus isolation/detection or serologically in Austria, Belgium, Croatia, Czech Republic, France, Germany, Greece, Hungary, Italy, the Netherlands, Poland, Serbia, Slovakia, Spain, Switzerland and the United Kingdom. The majority of published studies are country and/or host-based. This review aims to synthesize the current data on emergence (Table 1), dynamics and molecular epidemiology (Table 2, Figure 2) of USUV in Europe within the multidisciplinary “One Health” context.

**Figure 3:**
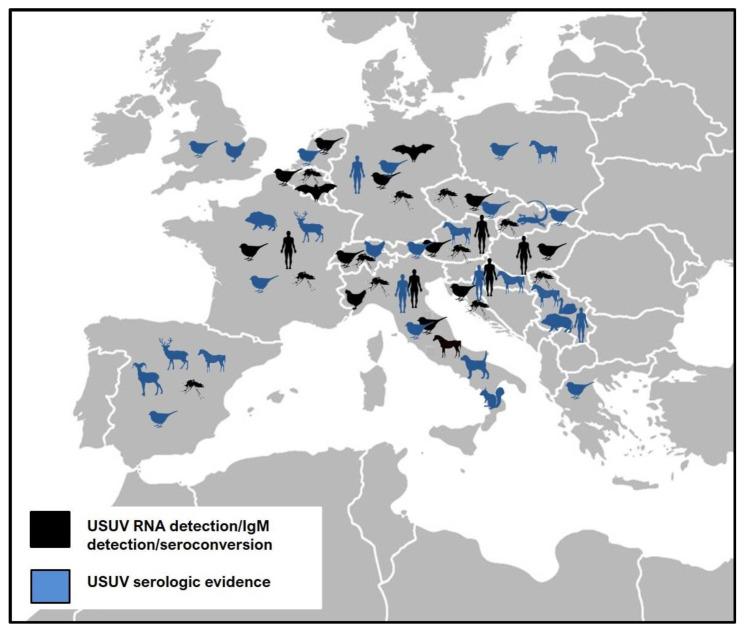
Geographic distribution of Usutu virus in Europe (clinical cases/RNA detection/seroconversion/serologic evidence).

The geographic distribution of USUV infections in European countries is presented in Figure 3.

## 2. Austria

USUV emerged in Austria in August 2001, causing increased wild bird mortality in Vienna and its surroundings. Eurasian blackbirds (*Turdus merula*) and great gray owls (*Strix nebulosa*) were affected [8]. In 2002, high numbers of avian deaths were recorded in the same region of the federal state of Lower Austria, while single die-offs were noticed in the federal states of Styria and Burgenland [38]. In 2001 and 2002, the only USUV Europe 1 lineage was found [62]. Monitoring of USUV activity using dead bird surveillance (2003–2005) found 92/177 birds were positive in 2003, 11/224 birds were positive in 2004 and 4/103 birds were positive in 2005, all of which were blackbirds [75]. To determine the prevalence of USUV antibodies, blood samples of 442 wild birds were collected during four consecutive years (2003–2006). To analyze dynamics of antibody titer and seroconversions, 86 individuals from a bird of prey rehabilitation center were bled before, at the peak, and after the 2005 USUV transmission season. While in 2003 and 2004 the proportion of seropositive wild birds was <10%, the percentage of seroreactors raised to >50% in 2005 and 2006, indicating a continuous increase in wild bird seropositivity [76]. In contrast, USUV seroprevalence was low (8.75%) in birds in Vienna zoo tested in 2006–2007 [77]. Between 2010 and 2015, a limited number of songbird carcasses (seven wild birds) were tested for USUV infection which all tested negative. Sequences obtained from two positive blackbirds in 2016 showed USUV Europe 2 lineage [34]. In 2017, a second wave of USUV-associated blackbird deaths was observed in eastern Austria with expanding of affected areas to the south and west in 2018. Except for one case of USUV Africa 3 lineage in 2017, Europe 2 remains the most prevalent genetic lineage [62]. During the outbreak, USUV RNA was found in 16/19 pools of *Cx. pipiens*/*Cx. torrentium* mosquitoes at sites of USUV linked blackbird mortality in Linz and Graz. In addition, USUV was detected in a pool of *Ae. japonicus*, which was the first report of natural infection of *Ae. japonicus* with USUV, suggesting that it may be involved in the epizootic USUV transmission in Europe [17]. Blood donations testing in eastern Austria in 2016 and 2017 found one and six USUV-positive donations, respectively [39]. In 2018, USUV RNA (Europe 2 and Africa 3 lineages) was detected in 18 blood donations. Sixteen donors remained asymptomatic, one developed a rash and one donor did not disclose information. One blood donor had a dual infection with West Nile virus (WNV) and USUV [35]. The presented data showed that USUV is widespread in Austria. The establishment of herd immunity in wild birds probably affects the epidemiology of USUV in Austria causing a decrease in bird mortalities in more recent years [76].

## 3. Belgium

In October 2012, USUV was detected in two birds (bullfinch; *Pyrrhula pyrrhula* and great spotted woodpecker; *Dendrocopos major*) that presented with neurological signs in the Meuse Valley, Belgium [40]. A passive surveillance program demonstrated a re-emergence of USUV in 2016 causing an epizootic among birds (mainly blackbirds) in Flanders. Coinfection with *Plasmodium* was detected in 99% of the dead passerine birds that were necropsied [10]. In one study, clinical and postmortem examinations were performed on five wild adult birds (three blackbirds, one robin; *Erithacus rubecula* and one house sparrow; *Passer domesticus*) which had been found moribund with severe neurological signs in private gardens in the province of Liège (2016). The detected USUV-Liege strain clustered within USUV Europe 3 lineage [36]. In addition, the virus was detected in overwintering *Cx. pipiens* pools collected in 2016 [1]. During the wildlife monitoring (birds and bats) in southern Belgium (2017–2018), USUV-RNA was detected in 69/253 birds from 15 species. Similarly, 2/10 bats were detected RT-PCR positive. Phylogenetic analysis showed that circulating strains in bats and mosquitoes belonged to USUV Europe 3 lineage, while one strain detected in a common chaffinch (*Fringilla coelebs*) showed a close genetic relationship with the European 1 lineage strains [20]. In 2018, USUV RNA was found in dead common scoters (*Melanitta nigra*) collected at five private parks located along the southern edge of the North Sea, straddling Belgium and the Netherlands. The common scoter is the first identified species from the Anseriformes family to be naturally susceptible to the infection by the USUV strain of Africa 3 lineage [78]. No horses were detected as positive for USUV so far, although virus endemization is highly suggested. There are no data on human USUV infections in Belgium.

## 4. Croatia

The first serologic evidence of USUV circulation in Croatia was reported in 2011 by detecting USUV neutralizing antibodies in two horses from north-west Croatia [22]. In 2012, neutralizing antibodies were detected in one human sample from a resident of eastern Croatia [79]. The first three cases of human neuroinvasive USUV infection were reported in Zagreb and its surroundings during the 2013 WNV outbreak [30,80]. From 2017 to 2020, a project on integrated (“One Health”) arbovirus surveillance in humans, sentinel animals and vectors was conducted (CRONEUROARBO). As a part of the project, three additional human cases of the neuroinvasive disease were detected in one north-western and two eastern Croatian counties during the large WNV outbreak in 2018 [32]. For the first time, in 2018, USUV was confirmed in the brain tissue of one dead blackbird from north-west Croatia. USUV-positive mosquito pools were detected in 2016 (*Ae. albopictus*), 2017 (*Cx. pipiens*), 2018 (*Cx. pipiens*) and 2019 (*Cx. pipiens*), respectively in north-western regions [15,32,81]. Four sequenced strains from a fatal human case, blackbird and two *Cx. pipiens* pools (2018, 2019) belonged to USUV Europe 2 lineage [32]. The sequences are deposited in the GenBank database under accession numbers MT891318-MT891321. The presented data suggest a continuous geographical spread of the virus and endemization of USUV in continental Croatia. So far, there are no reported USUV infections at the Croatian littoral.

## 5. Czech Republic

Between 2004 and 2006, a serosurvey was carried out in the Czech Republic in 54 domestic birds (geese and ducks bred on fishponds) and 391 wild birds representing 28 migratory and resident species. The birds were sampled in the South-Moravian fishpond ecosystem. Among 14 WNV-reactive samples tested for USUV, one coot (*Fulica atra*) had a higher titer against USUV, and another one could not be attributed to either of the two viruses [41]. In 2011, the Central European lineage of USUV was isolated from a dead blackbird in Brno. In addition, USUV RNA was detected in two other dead blackbirds in Brno during 2012 [64]. Mosquitoes (Culicidae) were collected at South-Moravian fishponds between 2010 and 2014 at three sites characterized by a reed bed ecosystem situated at the littoral zone of fishponds. One pool of *Cx. modestus* mosquitoes collected in 2013 (Mlýnský fishpond) proved positive for USUV RNA. This was the first detection of USUV in *Cx. modestus* mosquito species. Phylogenetic analysis demonstrated that the Czech USUV strain is closely related to Austrian and other Central European virus strains [42]. From 2017 to 2019, cadavers of blackbirds were collected in three cities and their surroundings: Ceske Budejovice (2017), Prague (2018) and Brno (2017–2019). Twenty (36%) blackbirds were positive for flavivirus RNA and subsequently confirmed as USUV by sequencing. Multiple USUV lineages (Europe 1, 2 and Africa 3) were detected in blackbirds in the southeastern region of the Czech Republic. In Prague, the increased mortality of the blackbird population in 2018 was likely associated with a single USUV lineage (Europe 3). USUV RNA (Europe 2 lineage) was detected in a pool of *Cx. pipiens* mosquitoes from the southern part of the country (South Bohemia), where no major mortality of birds has been reported. One pool of *Cx. modestus* mosquitoes (South Moravia, 2016) also tested positive for USUV RNA (Europe 2 lineage) [18]. There are no reports on human USUV infections. Detection of multiple genetic lineages in birds and mosquitoes indicates multiple USUV introductions in the Czech Republic.

## 6. France

Despite frequent detection of USUV in neighboring countries, the USUV has not been reported in France until 2015 [82]. In August and September 2015, unusual and grouped bird fatalities were observed in blackbirds in two regions in eastern France (Haut-Rhin and Rhône). USUV was confirmed in five birds subjected to molecular detection for flaviviruses. USUV strains from Haut-Rhin and Rhône departments were distinct from each other and arose from more than two independent introduction events. Phylogenetic analysis of the whole genome USUV isolates demonstrated that the viral strains detected in Haut-Rhin, which borders Germany, are genetically similar to USUV strains isolated in Central Europe. In contrast, the strain isolated from one blackbird in the Rhône shared the highest genetic homology with USUV strains detected in Spain [43]. In 2016, near Camargue, the first human case of USUV infection presented with idiopathic facial paralysis which was retrospectively identified by a flavivirus molecular survey of cerebrospinal fluid (CSF) samples [31]. USUV lineages Africa 2 and Africa 3 were detected in 11 *Cx. pipiens* pools collected in 2015 (Camargue), demonstrating the simultaneous occurrence of different strains within the mosquito population. These data reported, for the first time, detection of USUV in mosquitoes that concurrently accompanied the emergence of USUV in blackbirds and a human case during the period 2015–2016 [44]. A serological study conducted among wild ungulates (2009–2014) indicated the continuous circulation of USUV in southern France with seropositivity of 8.0% in wild boar and 1.0% in roe deer [26]. In addition, a serological survey conducted in 2009–2019 found low titers of USUV neutralizing antibodies in sentinel wild birds (magpie; *Pica pica*) in the Camargue area [82]. In 2018, high detection rates of USUV in birds was observed. The virus was detected from dead birds in 46 administrative districts, compared to only 4 in 2017 [83].

## 7. Germany

USUV was first detected within a mosquito-based surveillance program in Germany. During the entomologic survey conducted in 2010–2011, mosquitoes were collected at 11 sites in Germany. USUV was isolated in cell culture from one pool of *Cx. pipiens* mosquitoes trapped in August 2010 in Weinheim (upper Rhine valley). Phylogenetic analysis revealed a close relationship between the strain detected in a dead blackbird in 2004 and a USUV strain from Austria [13]. No increase in mortality of wild and captive birds was observed in Germany before 2011. In June 2011, blackbirds were frequently found dead around the cities of Mannheim and Heidelberg. In 2011, 223 birds were collected mainly in the upper Rhine valley and sent for USUV diagnostics. USUV RNA was detected in the organs of 86 birds from six species. Phylogenetic analysis revealed a close relationship with strain Vienna that caused high bird mortality in Austria in 2001 [9]. In the other study in the upper Rhine valley (2011–2013), 663 dead birds were collected and USUV RNA was detected in the organs of 209 birds for all three years. In the same study, a panel of 902 blood samples from migratory and resident birds was tested for USUV antibodies. Eight (0.89%) samples had USUV neutralizing antibodies [84]. These data showed that the virus spread in 2011 and caused epizootics among wild and captive birds in south-west Germany. One healthy blood donor showed IgM and IgG antibodies to USUV in 2012 (south-west Germany), while none of the patients with clinically suspected acute USUV infection tested positive for USUV antibodies during 2011–2012 [45]. Dead birds were screened for USUV from 2011 to 2015. A total of 230 specimens of 15 species (85.7% common blackbirds) from 132 different sites tested positive for USUV RNA [85]. In addition, mosquitoes collected from 2011 to 2016 throughout the country were screened for arboviruses. Two *Cx. pipiens* pools tested positive for USUV. Europe 3 and Africa 3 lineages were detected in Freiburg and Emsdetten, respectively [86]. In 2013, two dead bats (*Pipistrellus pipistrellus*) were found in south-west Germany, in a previously described USUV-endemic area. Both bat USUV strains belonged to USUV Europe 3 lineage and had 99.9% nucleotide and 99.8% amino acid identity [19]. In 2015, a new USUV strain was documented in two captive juvenile owls in the Zoological Garden Berlin (north-east Germany). Detected strain belonged to the Africa 2 lineage and differed from the other two strains circulating in Germany (USUV Europe 3 and USUV Africa 3 lineage, respectively) [87]. In 2016, numerous dead blackbirds were found around the city of Leipzig. Fourteen blackbirds and one great gray owl found in the zoo tested positive for USUV. Phylogenetic analysis showed the co-circulation of three different USUV strains in eastern Germany (Europe 3, Africa 2 and Africa 3-like). Furthermore, USUV was detected in *Cx. pipiens* in a region where no dead birds were reported (the city of Zeitz, 50 km distance from Leipzig) [65]. During the 2016 epizootics, acute asymptomatic infection (USUV Europe 3 lineage) was detected in one blood donor [63]. In 2017 and 2018, live wild and zoo birds were screened by RT-PCR and serological assays. Overall, 57 blood samples of the live birds and organ samples of 100 dead birds were positive. USUV Europe 2 lineage was detected for the first time in Germany and the spread of USUV lineages Europe 3 and Africa 3 towards northern parts of the country. USUV seroprevalence rates were high in eastern Germany in both years [66]. The presented data indicate that USUV has been circulating endemically causing periodic epizootics in Germany since 2011.

## 8. Greece

To date, there is only one published study on the seroprevalence of USUV in pigeons in Greece. Two samplings were performed in juvenile domestic pigeons (*Columba livia domestica*) after the 2010 and 2011 WNV epidemics. The one pigeon found positive for USUV-neutralizing antibodies was sampled from a pigeon pen in Veria city in November 2010 [46]. There are no data on USUV infections in humans and mosquitoes in Greece.

## 9. Hungary

In Hungary, passive surveillance of dead wild birds has been performed each year since 2003 as part of the avian influenza monitoring program. A selected subset of these bird specimens was also tested for USUV. Between 2003 and 2006, 332 dead birds belonging to 52 species were analyzed. In 2003 and 2004, all birds collected tested negative. In 2005, however, USUV was detected in organ samples of a blackbird found dead in Budapest. In 2006, a further six dead blackbirds found in urban areas of Budapest tested positive for USUV RNA, and the virus was isolated from organ samples of one bird. The nearly complete genome sequence of the Hungarian USUV strain shares a high similarity with that of the Austrian strain circulating since 2001 [67]. No antibodies to USUV were detected in birds in the Budapest zoological garden (2006–2007) [76]. Between 2010 and 2015, USUV caused sporadic cases of wild bird mortality, whereas in summer and autumn 2016 the number of cases considerably increased. In 2016, altogether 12 birds tested positive for USUV infection: one Eurasian jay (*Garrulus glandarius*), one starling (*Sturnus vulgaris*) and ten blackbirds. USUV sequences from Hungary collected in 2010, 2011 and 2015, respectively, grouped within USUV Europe 1 lineage, while a sequence from 2016 belonged to Europe 2 lineage [39]. The first human USUV infection in Hungary was described in 2018 in a patient with aseptic meningitis. USUV Europe 2 lineage was identified which showed 100% identity with a strain that was detected in a blackbird in 2016 [47].

## 10. Italy

In Italy, USUV was first detected in 1996 on a retrospective analysis of archived tissue samples from bird deaths in the Tuscany region [7]. In the summers between 2006 and 2008, USUV infection was confirmed in two free-living blackbirds and three captive owls in northern Italy. Phylogenetic analysis revealed 99.8–100% nucleotide identity of the Italian USUV strains to those from other Central European countries [88]. In 2007, a seroconversion was reported in sentinel chickens in the Ravenna province (northeastern Italy) [89]. Sentinel horses and chickens, wild birds and mosquitoes were sampled and tested for the presence of USUV within the WNV National Surveillance plan in 2008–2009. Seroconversion in chickens and horses as well as USUV RNA detection in birds proved that the virus has circulated in Tuscany, Emilia Romagna, Veneto and Friuli Venezia Giulia regions. In Veneto, USUV caused a severe blackbird off disease involving at least a thousand birds. USUV was also detected in a pool of *Cx. pipiens* caught in Tuscany. A higher seroprevalence in horses was found in 2008 (89.2%) compared to 2009 (7.8%) [21]. Data from the other study conducted in 2009 proved a co-circulation of USUV in wild birds and mosquitoes in northern Italy as well [48]. Mosquito, bird and human surveillance in the Emilia-Romagna Region in 2010 found USUV in *Cx. pipiens* and *Ae. albopictus* mosquitoes and birds, whereas no one patient with meningoencephalitis tested USUV positive [14]. The whole-genome sequences of 15 USUV strains isolated between 2010 and 2014 from mosquitoes and wild birds in the Emilia-Romagna and Lombardy regions (northern Italy) showed the circulation of USUV Europe 2 and Europe 4 lineages. Sequences from mosquitoes were mainly detected in *Cx. pipiens*, but also in *Ae. albopictus* and *Ae. caspius* [16]. In 2011–2012, a serologic survey was conducted in hunting dogs in the Campania region (South Italy) with an overall prevalence of 13.11% [24]. A total of 158 gray squirrels (*Sciurus carolinensis*) from northern Italy (Piedmont and Lombardy region) sampled between 2011 and 2013 showed USUV seropositivity of 3.2 to 3.8% [25]. In addition, in 2012–2013, 1.34% of wild birds in northwest Italy were seropositive for USUV [90]. Field data from an extensive entomological surveillance program found USUV-positive *Ae. albopictus* pools in the period 2009–2012, while all pools were negative from 2013 on [91]. In 2014, USUV was detected for the first time in *Cx. pipiens* mosquitoes in Liguria (northwestern Italy) [92]. To assess the potential role of ticks as carriers of USUV, 1721 ticks from 379 wild birds in northwestern Italy were collected between 2012 and 2014, however all samples tested negative, suggesting that *Ixodes* spp. ticks are not competent vectors for USUV [93]. First human cases of USUV neuroinvasive infection caused by USUV Europe 1 lineage were documented in 2009 [28,29,68]. A retrospectively conducted survey on CSF and serum samples collected between 2008 and 2011 in Modena showed the presence of USUV RNA in 1.1% and antibodies in 6.57% samples. Italian strains clustered together with Central European strains [94]. Additionally, four IgG seropositive blood donors were detected in 2012 in northeast Italy [95]. In 2014–2015, a very high USUV seroprevalence was found in forestry workers (18.1%) compared to blood donors (1%) in the area of the Po river valley (northern Italy) [96]. During 2016–2018, an unexpected high seropositivity (46.3%) for USUV was found in asymptomatic blood donors in the Lombardy region [97]. In 2017 and 2018, five blood donations tested in the Lazio region were USUV RNA positive with a circulation of different USUV strains (Europe 2, 3 and 4 lineages) [69]. In addition, six cases of human USUV infection (one with neuroinvasive disease, six with fever and one viremic blood donor with arthralgia and myalgia) were detected in northern Italy in 2018 [33].

## 11. Netherlands

In the Netherlands, USUV was first detected in 2016, when it was identified as the cause of an outbreak among blackbirds and great gray owls on postmortem examination by RT-PCR. USUV-positive birds were from sites located in the south-east of the Netherlands [49]. From November 2014 to May 2015, as part of avian influenza surveillance, serum samples collected from 250 resident and migratory birds were tested for flavivirus antibodies. USUV seroprevalence was found to be 2.8% [98]. Furthermore, from 2016 to 2018, 165 dead blackbirds were screened for USUV by RT-PCR of which 118 tested positive. Phylogenetic analysis revealed the co-circulation of USUV Europe 3 and Africa 3 lineages, with Africa 3 lineage most frequently detected [70]. In 2018, USUV Europe 3 lineage was found in two blood donors in the Netherlands [71].

## 12. Poland

Only two studies addressed the USUV seroprevalence in Poland to date. In 2006, USUV neutralizing antibodies were found in a black-headed gull (*Larus ridibundus*), representing the first seropositive case recorded in the country [50]. In the other study, tissue samples from birds and blood from horses and birds were collected from October 2012 to April 2013. Tissue samples were taken from 30 birds which died at the Wild Animal and Bird of Prey Breeding and Protection Center in Wroclaw. Seven birds showed central nervous system symptoms. Blood samples for serological testing were collected from 10 healthy goshawks trapped in the field and four sick birds treated at the center. Horses included in the survey were from different farms throughout the country. USUV was not detected in any of the bird tissue samples tested, however USUV-neutralizing antibodies were found in one goshawk (*Accipiter gentilis*; 7.14%) and 27.98% of horses [51]. So far, USUV has not been detected in humans and mosquitoes in Poland.

## 13. Serbia

In Serbia, USUV was serologically confirmed in 0.3% (1/349) horses tested during the period 2009–2010, and 1.3% (4/318) wild boars tested during the period 2011–2012 in Vojvodina Province [52,53]. Additionally, USUV RNA was confirmed in 0.4% *Cx. pipiens* pools from Vojvodina in 2014 [72] and 0.93% *Cx. pipiens* pools in 2015 [54], as well as in 2.75% *Cx. pipiens* pools in 2017. One isolate from 2014 was genotyped as USUV Europe 1 lineage [72] and two isolates from 2017 as USUV Europe 2 lineage [73]. Moreover, USUV antibodies were detected in 5% human serum samples in South Bačka District (Vojvodina) tested during 2015 [54]. So far, USUV is still not detected in wild birds in Serbia, but serological confirmation of USUV circulation in wild birds was detected in one mute swan (*Cygnus olor*) out of seven wild birds tested positive for flavivirus (WNV ELISA) antibodies in 2012 [99].

## 14. Slovakia

There are few data on the prevalence of USUV in Slovakia. One study assessed the seroprevalence of flavivirus infections in horses and birds. A total of 145 horse serum samples collected in 2013 and 109 bird serum samples collected from 2010 to 2014 were tested for flavivirus antibodies. None of the tested horses was USUV seropositive, while neutralizing antibodies were present in a pooled sample from four Eurasian great tits (*Parus major*) in Levice County [23]. In the other study, USUV-neutralizing antibodies were found in 26.8% of the serum samples of green lizards (*Lacerta viridis*) captured during the period 2017–2018 in the Slovak Karst National Park [27]. In 2018, mosquito sampling was conducted in the south-western part of the country (Komárno district, Nitra region). Seven pools of *Cx. pipiens* yielded positive USUV sequences, with the minimal prevalence of 0.25% in all tested mosquitoes. Detected strains belonged to USUV Europe 2 lineage [55]. 

## 15. Spain

USUV was discovered for the first time in Spain in 2006 in a pool of *Cx. pipiens* mosquitoes in Catalonia, from the area where different common migratory and sedentary birds feed and nest [56]. In 2008 and 2009, the virus was detected in *Cx. perexiguus* pools collected in southern Spain. Phylogenetic analysis showed that the Spanish strains detected in 2006 and 2009 were more related to the African USUV isolates than Central European isolates [74]. In 2011, an epidemiological survey was conducted to determine the flavivirus seroprevalence in waterfowl used as decoys and wild raptors in Andalusia (southern Spain), the region considered to have the highest risk of flaviviruses circulation in Spain. The frequency of USUV-positive decoys ranged from 4.4 to 5.9% [57]. A flavivirus surveillance program implemented in partridges (*Alectoris rufa*) and pheasants (*Phasianus colchicus*) between 2011 and 2012 showed a seroprevalence for USUV of 10% with higher seropositivity for pheasants (54%) than for partridges (7%) [58]. In addition, monitoring of horses from localities near wetlands as "sentinel" hosts (Mallorca Island, 2011–2012) showed USUV seroprevalence of 1.2% [59]. In 2012, USUV RNA was detected in two song thrushes (*Turdus philomelos)* in southern Spain who died of encephalitis [100]. An extensive study on WNV transmission was conducted in the provinces of Huelva, Cádiz and Sevilla (southern Spain) in different migrant and resident birds captured during 2013. A serum sample from one blackbird neutralized both USUV and WNV with higher titer to USUV compared to WNV [101]. From 2003 to 2014, exposure to USUV was assessed in wild ruminants. Serum samples from free-living and farmed red deer (*Cervus elaphus*), fallow deer (*Dama dama*), mouflon (*Ovis aries musimon*) and roe deer (*Capreolus capreolus*) were tested with the seroprevalence of 0.1–0.2% according to the bioregion [102]. A serosurvey conducted in feral pigeons (*Columba livia* var. *domestica*) and captive zoo birds from Córdoba (southern Spain) between 2013 and 2014 found 3.6% USUV-seropositive animals [103]. Detection of USUV in mosquitoes, birds and seropositive horses and wild ruminants indicate that the virus circulates in Spain. However, no human cases of USUV infection are reported so far.

## 16. Switzerland

The emergence of USUV among bird species in Switzerland was first recognized in late summer of 2006 in a zoological facility, and it repeated subsequently in 2007 and 2009 with birds dying either acutely or with neurological disturbances. Almost all affected birds were found within Zurich Zoo, except one blackbird, which was found dead 15 km outside the zoo. The affected birds were primarily wild and captive Passeriformes and Strigiformes. Partial nucleotide sequence comparisons revealed >99% identity between the viruses that emerged in Zurich (2006), in Vienna (2001) and in Budapest (2005) [60]. The house sparrows were the most affected species in the Swiss outbreak compared to the Austrian outbreak where more blackbirds were dying of USUV infection [8]. In addition, between October 2006 and August 2007, sera from different captive birds in Zurich Zoo were tested, demonstrating low degree of USUV exposure (5.3%). USUV-specific antibodies were detected in a marabou stork (*Leptoptilos crumeriiferus*), ruddy shellduck (*Tadorna ferruginea*), red-breasted goose (*Branta ruficollis*), Humboldt penguin (*Spheniscus humboldti*), laughing kookaburra (*Dacelo novaeguineae*), steamer duck (*Tachyeres pteneres*) and domestic chicken (*Gallus gallus domesticus*). Six USUV-seropositive samples from the Basel Zoo originated from greater flamingos (*Phoenicopterus ruber)* [77]. Subsequently, 258 human cerebrospinal fluid samples collected between 2015 and 2017 for routine clinical care in a tertiary level hospital in Geneva were tested for USUV by RT-PCR, but no samples tested positive [104]. In 2011 and 2012, USUV was found in mosquitoes from Ticino [105], and recently confirmed in the same area [106]. USUV-positive mosquito pool of *Cx.pipiens*/*Cx. torrentium* shared the highest sequence similarity with USUV isolated from *Cx. pipiens* in northern Italy in 2010 [14]. Since 2006, in Switzerland the USUV infection affected animal species only, with no human cases reported.

## 17. United Kingdom

Very few published studies addressed the presence of USUV in the UK. A serologic survey on 91 serum samples from several bird species and poultry (blackbird, carrion crow, magpie, robin, turkey) collected in 2001 and 2002 showed that 49 of them (53.8%) were positive for USUV neutralizing antibodies. USUV has probably been introduced into UK-resident birds from migrant birds via indigenous mosquitoes [61]. USUV antibodies were also confirmed in serum samples of sentinel raised chickens on an English farm in Cambridgeshire in 2004 [107]. A retrospective targeted surveillance of 201 birds examined postmortem between 2005 and 2011, submitted from different sites across England and Wales, showed no evidence of USUV [108]. Subsequently, a large mosquito survey on 11 different species (*Anopheles claviger*, *An. maculipennis* s.l., *An. plumbeus*, *Coquillettidia richiardii*, *Ochlerotatus caspius*, *Oc. dorsalis*, *Oc. detritus*, *Oc. flavescens*, *Cx. pipiens* s.l., *Cx. torrentium*, *Cx. modestus* and *Culiseta annulata*) from the North Kent Marshes in 2013, identified no presence of USUV RNA in any of the samples [109]. There were only few data on the serologic evidence of USUV infection in birds and chickens in the UK, while still there are no data on the USUV detection in birds, humans or mosquitoes. Because mosquito densities in the UK are relatively low compared with warmer countries, such as those in southern Europe, the likelihood of successful transmission of the USUV to local birds from migratory birds might be expected to be low [61].

## 18. Conclusions

So far, USUV infections were reported in Europe, Africa and the Middle East [110]. Although in Africa, the virus was identified in the late 1950s, very limited published data are available, while in Europe, the virus emergence has prompted numerous studies with robust epidemiological surveillance programs. Bird mortality caused by USUV was not reported in Africa [3], while in Europe, USUV was shown to be highly pathogenic for several bird species, especially blackbirds, great gray owls and house sparrows [12,38,111]. In Africa, only two USUV-related human infections were reported which presented with fever, rash and jaundice [3]. In Europe, neurotropism of USUV for humans was reported for the first time in both immunocompromised and immunocompetent patients [28,29,30,32,80]. While a silent circulation of USUV was reported in 1996–2001, continuous USUV activity confirmed by detection and/or serologic evidence of USUV in birds, humans, horses, mosquitoes and bats since 2001 indicates the endemization in many Southern and Central European countries. However, due to a limited number of human USUV infections, zoonotic potential and clinical relevance of USUV needs to be further investigated. Circulation of different USUV lineages, including European and African, suggests multiple introduction events to Europe from Africa. The Europe 2 lineage is the most commonly detected USUV lineage in the European countries [16,32,55,73]. The USUV Europe 3 lineage was predominantly circulating in southern Belgium in 2017 and 2018 [20]. Recent studies from Germany [66] and the Netherlands [70] showed an increase in the USUV Africa 3 lineage detection during the same period. The genetic diversity of European lineages is most likely shaped by enzootic maintenance (*in situ* evolution) rather than by extensive migration, while African lineages are driven mostly by extensive migration and repeated introduction of viral variants from different geographic origins [37]. In addition to data from the published articles, the sequences retrieved from the GenBank suggest for certain European countries, a broader host range as well as USUV lineage diversity within a specific host. 

The integrated veterinary and human surveillance system (“One Health”) based on the virus detection in mosquitoes, migratory and resident birds as well as horses and poultry was proved to be useful for estimating the public health risk for other flaviviruses such as WNV. This enables the effective and timely control of the diseases in humans [112,113,114]. Since spreading trends of USUV are likely to continue, continuous multidisciplinary interventions in accordance with the “One Health” concept, should be conducted to increase the awareness of USUV and implement appropriate monitoring and prevention methods for this emerging arboviral infection. 

## Figures and Tables

**Table 1:** Emergence of Usutu virus (USUV) in Europe: clinical cases/seroconversion/RNA detection/serologic evidence. In Europe, USUV emerged in 1996 in the Tuscany region (Italy) when it was detected by retrospective analysis of archived tissues from dead birds. In mosquitoes, USUV was discovered for the first time in 2006 (Spain) in a pool of *Cx. pipiens*. Seroconversion in horses was reported in 2008 (Italy), following the first two human clinical cases in 2009. In 2013, the virus was detected in two dead bats (*Pipistrellus pipistrellus*) in Germany. Until 2018, USUV infections were reported in 16 European countries.

Country	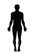	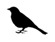	* 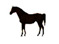 *	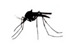	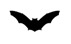	Reference
Austria	2016*	2001		2017		[8,17,37,38,39]
Belgium		2012		2016	2017	[1,20,40]
Croatia	2012	2018	2011	2016		[15,22,30,32]
Czech Republic		2004		2013		[41,42]
France	2016	2015		2015		[31,43,44]
Germany	2012*	2011		2010	2013	[9,13,19,45]
Greece		2010				[46]
Hungary	2018	2005				[34,47]
Italy	2009	1996	2008	2009		[7,21,28,29,48]
Netherlands		2016				[49]
Poland		2006	2012			[50,51]
Serbia	2015	2012	2009	2014		[52,53,54]
Slovakia		2010				[23,55]
Spain		2011	2011	2006		[56,57,58,59]
Switzerland		2006				[60]
United Kingdom		2001				[61]

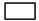
Clinical cases/RNA detection/seroconversion;
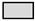
serologic evidence; *asymptomatic blood donors/blood donations.

**Table 2:** Molecular epidemiology of Usutu virus (USUV) in Europe. USUV Europe 2 lineage is the most prevalent genetic lineage detected in birds, mosquitoes and humans. In birds, Europe lineage 1, 3–5 and Africa 2 and 3 lineages were detected as well. Europe 3 and 4 and Africa 2 and 3 lineages were detected in mosquitoes. In bats, only Europe 3 lineage was documented so far.

Country	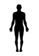	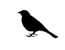	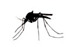	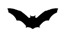	Reference
Austria	Europe 2* Africa 3*	Europe 1,2 Africa 3	Europe 2**		[34,35,62,63]
Belgium		Europe 1,3 Africa 3	Europe 3	Europe 3	[20,36,62,63]
Croatia	Europe 2	Europe 2	Europe 2		[32]
Czech Republic		Europe 1,2,3 Africa 3	Europe 2		[18,42]
France	Africa 2	Europe 3	Europe 2 Africa 2,3		[31,44]
Germany	Europe 3	Europe 2,3,5 Africa 2,3 Africa 3-like	Europe 3 Africa 3	Europe 3	[9,19,63,64,65,66]
Hungary	Europe 2	Europe 1,2			[34,39,47,67]
Italy	Europe 1,2*,3*,4*	Europe 2,4	Europe 2,4		[16,29,63,68,69]
Netherlands	Europe 3	Europe 3 Africa 3			[70,71]
Serbia			Europe 1,2		[72,73]
Slovakia			Europe 2		[55]
Spain		Africa 2	Africa 2		[1,74]
Switzerland		Europe 1			[60]

*Blood donors/donations; **based on nucleotide sequences retrieved from the GenBank.
